# Exploring the Immigrant Health Paradox Among the Vietnamese Population in the United States

**DOI:** 10.3390/healthcare14030354

**Published:** 2026-01-30

**Authors:** Tran Nguyen, Gia-Thien Nguyen, Raymond Chong, Yoon-Ho Seol

**Affiliations:** 1 School of Public Health, Augusta University, Augusta, GA 30912, USA; yseol@augusta.edu; 2 Medical College of Georgia, Augusta University, Augusta, GA 30912, USA; nguyen267@hotmail.com; 3 College of Allied Health, Augusta University, Augusta, GA 30912, USA; rchong@augusta.edu

**Keywords:** Vietnamese Americans, Vietnamese immigrants, immigrant health paradox, mental health, depression, perceived financial needs

## Abstract

**Background**: The term *immigrant health paradox* describes how immigrants often have better health outcomes than their American-born counterparts. While existing literature treats this phenomenon as broadly generalizable, emerging research indicates that its expression varies across cultural and migration contexts. Understanding how the immigrant health paradox may appear across specific ethnic groups requires research that maps variation rather than assumes uniformity. **Objectives**: This study seeks to describe patterns, explore variation by nativity, and identify factors associated with well-being among the Vietnamese population in the United States (US). By focusing on descriptive trends and contextual influences, the study aims to generate new insights into how the paradox may manifest—or diverge—in the Vietnamese context. **Methods**: We conducted an online survey asking participants about their depressive disorders, physical and mental health status, demographics, socioeconomic status, social networks, and experiences with daily discrimination. Descriptive statistics were used to describe the study sample. Linear regression and ordinal logistic regression were performed to explore the relationships. **Results**: In this exploratory analysis, we did not observe indications of the Vietnamese immigrant health paradox. Material factors, especially perceptions of financial needs, as well as psychological factors, were somewhat associated with how Vietnamese people living in the US assess their health. **Conclusions**: The absence of the Vietnamese immigrant health paradox in the US underscores the need for nuanced health models that reflect diversity within immigrant groups. Their experiences reveal how migration histories, structural barriers, and racialization shape health outcomes in ways that differ from expectations.

## 1. Introduction

The United States boasts one of the most diverse populations in the world, shaped by centuries of migration and ongoing demographic changes. Immigrants contribute significantly to this diversity, bringing unique cultural practices, health beliefs, and migration stories that impact their well-being. Among these groups, Vietnamese immigrants stand out for their distinct migration patterns and cultural backgrounds, which shape their specific health needs and strengths.

### 1.1. Vietnamese Immigrants

Recently, Asians have become the fastest-growing major racial or ethnic group in the United States (US) [1]. The Vietnamese population ranks as the fourth-largest Asian ethnic group, following the Chinese (excluding Taiwanese) with 5.5 million, Indians with 5.2 million, and Filipinos with 4.6 million, while surpassing Koreans with 2.0 million and the Japanese with 1.6 million [2]. In 2023, it is estimated that 2.3 million Vietnamese people lived in the US, representing roughly 0.7% of the total US population. Due to historical events related to the Vietnam War, this population is diverse, comprising various immigration and citizenship statuses and different nativity backgrounds [3].

Before the Vietnam War ended in 1975, only a few thousand Vietnamese lived in the United States. These individuals were primarily spouses of American civilians or military personnel who had served in South Vietnam, as well as students and members of the Vietnamese diplomatic corps [4]. The “Fall of Saigon” led to one of the most significant and most prolonged refugee movements in modern history. When the South Vietnamese government collapsed, the new Communist regime began targeting former South Vietnamese military officers, government officials, intellectuals, religious leaders, and their families. As a result, hundreds of thousands of Vietnamese fled the country in 1975. From 1975 into the 1980s, as the humanitarian crisis and population displacement in the Indochina region (Vietnam, Cambodia, and Laos) worsened, the US welcomed more Vietnamese refugees and their families through the Orderly Departure Program (ODP) [5].

The fall of Saigon did not merely mark the end of a war; it initiated a prolonged period of political persecution that drove sustained migration. The Communist government’s policies toward former South Vietnamese officers and their families created conditions of fear, imprisonment, and economic marginalization, making emigration a necessity for many. In 1989, President Ronald Reagan signed a decree allowing Vietnamese ex-prisoners—those who had spent at least three years in re-education camps in Communist Vietnam—and their families to enter the US through a subprogram of the ODP known as the Humanitarian Operation (HO), which continued until the end of 1999 [6,7]. Since then, Vietnamese have been able to settle in the US for family reunification, particularly for the descendants of American citizens [4].

According to the Pew Research Center, 60% of Vietnamese living in the US are foreign-born, representing the first generation of Vietnamese Americans, while 40% are US-born, encompassing second- and third-generation Vietnamese Americans [8]. In this context, the term “Vietnamese immigrants” refers to the foreign-born Vietnamese, including refugees and immigrants who arrived due to the Fall of Saigon evacuation or through the ODP or HO. Vietnamese immigrants face unique challenges that significantly impact their well-being, including trauma from the war, family separations, torture endured in re-education camps, discrimination, and difficulties adjusting to a new country [9]. Their well-being is further affected by the intergenerational transmission of trauma [10,11]. Notably, high rates of clinical depression have been reported among this group, underscoring the need for targeted research and interventions [12].

Social support from family and friends is crucial for the mental health and successful integration of Vietnamese immigrants. Vietnamese immigrant families often maintain strong, multi-generational ties that extend beyond the household, providing both emotional and practical assistance. Family collectivism and resource sharing are important strategies for addressing economic and social challenges [13]. While family is typically the primary source of support, friendships—whether among relatives or non-relatives—are becoming increasingly significant, particularly when family support is limited or strained. Community organizations and social groups play a vital role in resolving disputes and facilitating adaptation [14]. High levels of support from family and friends act as a buffer against acculturative stress, depression, and loneliness, especially among older adults and those facing language barriers.

### 1.2. Immigrant Health Paradox

Individuals who immigrate often settle in diverse communities and adapt to various social and cultural norms, dietary choices, job opportunities, educational resources, and health-related issues [15]. However, immigrants frequently face challenges such as low health literacy, lack of insurance, limited English proficiency, and difficulties in communicating with healthcare providers. Experiences of discrimination also affect their interactions within the host society. Additionally, socioeconomic factors—including policies, housing availability, employment access, and infrastructure—significantly impact immigrants’ overall health [16,17].

Health disparities among different immigrant groups are well-documented. Despite numerous obstacles, research on the mental and physical health of these populations reveals an interesting trend: immigrants often report better overall health than their US-born counterparts. Generally, first-generation immigrants—those born abroad and relocate to the US—exhibit healthier outcomes and behaviors than their second-generation (born in the US to immigrant parents) and third-generation (born in the US to parents who are US-born but have immigrant roots) counterparts. This phenomenon is known as the *immigrant health paradox* [18,19,20].

The trend of the *immigrant health paradox*, however, tends to decline with longer durations of residence in the US and across subsequent generations. Research on immigrant health highlights the complexity and variability of the immigrant health paradox. In their systematic review of Immigrant and Hispanic Paradoxes, Teruya and Bazargan-Hejazi emphasize that the reported health advantages of immigrants are often inconsistent and influenced by methodological limitations, such as underreporting of deaths and incomplete data [19]. They note that the paradoxical health benefits vary significantly across different racial, ethnic, gender, and age groups. Their review identifies several key predictors that affect whether immigrants retain or lose their initial health advantages, including acculturation, health behaviors, acculturative stress, age at arrival, documentation status, and length of U.S. residence. Similarly, Markides and Rote describe broader trends related to the healthy immigrant effect, indicating that immigrants in major destination countries like the United States, Canada, and Australia typically arrive with better health and lower mortality rates, primarily due to positive health selection. However, they also observe a gradual convergence in health over a span of 10 to 20 years after arrival, leading to an increase in comorbidities and disabilities among older immigrants [21]. Together, these reviews highlight that while many immigrants start with strong health profiles, various factors—including acculturation pressures, socioeconomic challenges, structural barriers, and aging-related vulnerabilities—gradually diminish this initial advantage across different immigrant populations.

### 1.3. Vietnamese Immigrant Health Paradox

Several studies have documented that, as a group, Asian immigrants often report better physical and mental health than the US-born Asian population, particularly shortly after their arrival [22,23,24,25,26,27,28]. However, this trend is not uniform across all Asian subgroups. Some research suggests that certain populations may not benefit from this health advantage or may even experience worse health outcomes for specific conditions. Methodological issues, such as grouping diverse Asian subgroups, can obscure genuine disparities [29]. In the US, immigrants from approximately 30 Asian countries are categorized simply as “Asian,” without distinguishing between their nationalities. Risk factors and health outcomes can vary significantly across these immigrant subpopulations by country of origin. There is a notable gap in the literature regarding the unique risk and protective factors for each immigrant subpopulation. Recently, the National Institutes of Health has called for research that focuses on distinct immigrant subpopulations by country of origin, rather than treating them as part of a broader racial or minority category (for example, studying Koreans, Vietnamese, or Cambodians, rather than Asian Americans as a whole). Studies that compare different populations are encouraged to highlight immigrant-specific phenomena rather than making general comparisons between immigrants and non-Hispanic whites or the broader US population [30].

Despite being the fourth-largest Asian subgroup in the US, there is limited knowledge about the health of the Vietnamese population. Unlike many other Asian groups whose immigration was primarily driven by economic or educational opportunities, the initial waves of Vietnamese immigrants arrived as refugees following the end of the Vietnam War in 1975. These individuals were forced to flee political upheaval, resulting in distinct demographic, economic, and political characteristics. Vietnamese immigrants tend to be less economically advantaged and more politically conservative than other Asian American groups. They also generally have lower educational attainment and income levels, but show strong civic engagement [8,31].

The Vietnamese population plays a vital role within the broader Asian American population. Their unique immigration experiences, combined with trauma from the Vietnam War, significantly influence their health behaviors and outcomes. Studying the immigrant health paradox is essential for understanding whether Vietnamese immigrants—despite facing socioeconomic and historical challenges—arrive in the US with better health outcomes than expected but experience declining health over time. This paradox reveals hidden strengths and vulnerabilities that shape public health policy and support systems for immigrants. By focusing on this specific subgroup, we can gain a deeper understanding of the health challenges faced by Asian Americans, moving beyond a generalized “immigrant” label to uncover particular factors that influence their health.

### 1.4. Study Objectives

The purpose of this study is to explore health patterns among Vietnamese immigrants in the United States, with a focus on understanding how the immigrant health paradox may appear in this population. Specifically, the study aims to (1) describe patterns related to the immigrant health paradox among Vietnamese individuals in the US, (2) explore variation in well-being by nativity within the Vietnamese population, and (3) identify contextual and demographic factors associated with well-being among Vietnamese people. The findings are intended to inform future hypothesis-driven research and contribute to a more nuanced understanding of immigrant health dynamics.

## 2. Materials and Methods

This study employed a quantitative cross-sectional convenience sample to explore whether the immigrant health paradox is present among the Vietnamese population in the United States (US). It also identifies factors associated with the Vietnamese population’s well-being, defined as depressive disorder score, physical health, and mental health. The Augusta University Institutional Review Board (IRB) exempted this study from ethical review and approval because it poses minimal or no risk to participants (Exemption Category #2a, Project #1730410). An online survey was conducted using Qualtrics XM Platform (Qualtrics, Provo, UT, USA) in both English and Vietnamese to assess participants’ physical and mental health status, demographics, socioeconomic status, social networks, acculturative stress, and experiences of daily discrimination. The survey also included the Patient Health Questionnaire (PHQ-2), a two-item screening tool for depressive disorders. The Supplementary Materials presents the study’s instrumentation.

The study was promoted through various social media platforms, including Instagram, Facebook, Twitter, LinkedIn, Reddit, and online Vietnamese forums, as well as through the authors’ networking and word-of-mouth referrals. Recruitment methods included: (1) contacting Vietnamese businesses, clubs, organizations, and religious centers to request posting of recruiting materials on their social media platforms and distributing them to employees and members; (2) privately messaging and texting individuals within Vietnamese networks; (3) sharing information about the study on team members’ social media accounts and in Vietnamese online forums; and (4) utilizing targeted advertisements aimed at Vietnamese on platforms such as Facebook, Instagram, Reddit, and LinkedIn. The recruitment materials consisted of: (1) an invitation letter with a brief description of the study; (2) social media posts; and (3) email messages containing a synopsis of the study and a link to the survey’s consent form.

Individuals interested in participating had to digitally sign a consent form before accessing the study survey. Those who consented were then screened with three questions to determine their eligibility: whether they are at least 18 years old, of Vietnamese origin or ancestry, and either American citizens or permanent residents (green card holders). Only those who answered “yes” to all three questions were permitted to proceed to the complete study survey.

The current study focused on three dependent variables: participants’ depressive disorder score, physical health, and mental health. We assessed depressive disorders using the Patient Health Questionnaire-2 (PHQ-2), which evaluates the frequency of depressed mood and anhedonia over the past two weeks [32]. The PHQ-2 consists of two questions that ask participants how often they experienced (1) little interest or pleasure in doing things and (2) feelings of being down, depressed, or hopeless. Each question is scored as follows: 0 for “not at all,” 1 for “several days,” 2 for “more than half the days,” and 3 for “nearly every day.” Total scores on the PHQ-2 range from 0 to 6. A score between 3 and 6 indicates a major depressive disorder, while a score below 3 suggests a minor depressive disorder. A score of zero indicates that no depressive disorder is present.

The participants’ self-reported physical and mental health were assessed using survey items that asked them to rate their health on a 4-point Likert scale, ranging from poor/fair to excellent.

The independent variables included participants’ nativity, demographic characteristics, socioeconomic status, and material and psychosocial factors [22,33,34]. Nativity was classified as either non-US-born or US-born. The demographic variables included age (in years), gender (male or female), and marital status (married/living with a partner, single, widowed, or divorced). Socioeconomic status was comprised of factors such as age upon arrival in the US (in years), duration of residence in the US (in years), English proficiency (not at all, not well, well, or very well), Vietnamese proficiency (not at all, not well, well, or very well), employment status (non-employed or employed), education level (less than 12 years, 12 years or high school equivalent, some college, college graduate, or post-college), and household income (in US dollars). Material factors included health insurance status (no or yes) and perceived financial needs (insufficient, sufficient, or beyond sufficient). Psychosocial factors encompassed six items measuring social support from family and friends and three items assessing experiences of discrimination. For details, see the Supplementary Materials.

All measures of physical and mental health status, socioeconomic status, social networks, acculturative stress, and daily discrimination were derived from the National Latino and Asian American Study (NLAAS) question sets [33]. The NLAAS integrates items adapted from established national health and social surveys and has undergone extensive development procedures to ensure cultural and linguistic appropriateness for Latino and Asian American populations, including Vietnamese respondents. The NLAAS instrument has been documented for the validity and applicability with various Asian populations [22,34].

The NLAAS-derived questions used in this study were translated from English to Vietnamese by a certified Medical Translation Service [35], approved by the IRB. The translated items were then reviewed by the first author, who is fluent in both English and Vietnamese, to ensure cultural relevance, accuracy of terminology, and conceptual equivalence. This step ensured that linguistic nuances and culturally embedded meanings were preserved in the Vietnamese version of the instrument.

A pilot survey was conducted with five individuals: two fluent in English, two fluent in Vietnamese, and one fluent in both languages. We evaluated the clarity, relevance, and comprehensibility of the questions. The survey was open to the public from 2 January 2022 to 30 April 2022. To prevent potential fraudulent responses in the online survey, we enabled Qualtrics’ fraud-detection feature. We received 38 responses in Vietnamese and 104 in English.

The datasets were exported from Qualtrics in Excel format and underwent a structured data cleaning process before analysis. Initial exploratory checks were conducted to identify anomalies, such as out-of-range values, inconsistent response patterns, and irregular completion times. Qualtrics’ post-collection data tools were utilized to flag and remove cases that failed embedded attention-check items or exhibited survey durations that were suspiciously short or long, suggesting low-quality responses. Additional screening was performed to identify and exclude cases with missing responses on key analytic variables. This included instances where more than half of the demographic characteristics, PHQ-2 items, self-reported physical and mental health status, or nativity were missing. Logical consistency checks were applied across related items to detect implausible or contradictory responses, and cases were removed when inconsistencies could not be resolved. After applying these criteria, the final analytic sample consisted of 99 respondents: 32 in the Vietnamese version and 67 in the English version, combined into a single dataset for analysis.

Descriptive statistics were used to characterize the study sample. As the variable representing depressive disorders is a continuous one ranging from zero to 6, we fitted linear regressions to explore the association between depressive disorders and various exploratory factors. To explore the relationship between physical and mental health and exploratory factors, we conducted ordinal logistic regressions, given that both physical and mental health variables are ordinal.

Multicollinearity among the independent variables explored in this study is a well-documented issue in statistical analysis [36,37,38,39,40]. These variables are often conceptually and empirically interconnected. For instance, nativity may correlate with language proficiency, which, in turn, can influence socioeconomic status and exposure to discrimination. Socioeconomic indicators, such as income and education, are closely linked to material conditions and perceived financial strain. Levels of social support and experiences of discrimination frequently shape psychosocial stressors. Including all these variables in a single model can result in overlapping information, inflated standard errors, and unstable coefficient estimates [41,42,43]. To address these statistical concerns, we developed four regression models for each dependent variable. The first model included nativity as the independent variable, while the second incorporated demographics and socioeconomic status. The third model focused on material factors, and the fourth addressed psychological factors. Variance inflation factors (VIF) were examined to assess multicollinearity among independent variables for each regression model. VIF values ranged from 1.94 to 7.36, with a mean VIF of 4.87, indicating no evidence of severe multicollinearity and suggesting that the predictors were sufficiently independent for reliable estimation. The data analysis was performed using STATA version 18 (StataCorp LLC, College Station, TX, USA), with a significance threshold of 95% (*p*-value < 0.05).

## 3. Results

### 3.1. Descriptive Statistics—Sample Characteristics

Among the participants, 35% were born in the US, while 65% were born elsewhere. Those not born in the US had an average residency of 22 years and arrived at a mean age of 22. The mean age of all participants was 39, with two-thirds identifying as female. Most participants spoke English well (75%) and held a college degree or higher (62%). Additionally, 75% of the participants were employed, and 70% had private health insurance. The mean reported income was $87,000, with a range from $5000 to $400,000.

Regarding marital status, 60% were married or living with a partner, 31% had never married, and 7% were widowed. Sixty-seven percent (70%) reported having sufficient financial resources, while 24% felt they had more than enough, and 9% reported having insufficient resources. The study also found that 26% of participants had no depressive disorders, 32% had minor depressive disorders, and 42% had major depressive disorders.

The majority of participants rated their physical and mental health as good (57% and 47%, respectively) or very good (25% and 24%, respectively). About 8% described their physical health as poor or fair, and 13% rated their mental health as poor or fair.

Most participants did not report experiencing discrimination and maintained regular communication with friends and family for support. However, among those with limited English proficiency, 93% reported difficulties in socialization, and 72% experienced discrimination. Table 1 presents the characteristics of the study sample.

### 3.2. Multivariable Regression Analysis

Table 2, Table 3 and Table 4 present the results of the regression analyses. The findings demonstrate that nativity was not significantly associated with depressive disorders, physical health, or mental health. Therefore, our exploratory findings suggest that the *immigrant health paradox* is not present among the Vietnamese population in the US.

Table 2 presents the results of the linear regression analyses, which identified several factors significantly associated with depressive disorder scores. These factors include the length of time individuals have lived in the US, the age at which they arrived in the US, their perceived financial need, and the frequency of poor service experiences. Specifically, a longer duration of residence in the US, an older age at arrival, and more frequent encounters with inadequate services are associated with higher scores for depressive disorders. Conversely, a positive perceived financial need is associated with lower scores for depressive disorders.

The ordinal logistic regression model fits were tested, and the data were well fit, as indicated by a significant likelihood-ratio chi-square test with *p*-values less than 0.05 (ranging from 0.001 to 0.023). The model explained a substantial portion of the variance in the ordered outcome, with Pseudo R^2^ values ranging from 0.199 to 0.492. These results indicate that the predictors collectively make a meaningful contribution to predicting the ordinal dependent variable.

Table 3 presents the results of the ordinal logistic regression analysis of exploratory factors associated with participants’ self-rated physical health. The findings indicate that English proficiency, perceived financial need, frequency of communication with family, and level of openness to friends are all significantly associated with self-rated physical health. These results suggest that individuals with higher English proficiency, a favorable view of their financial situation, more frequent communication with family, and greater openness to friends are more likely to report better physical health.

Table 4 presents the results of the ordinal logistic regression analysis exploring factors related to participants’ self-rated mental health. The findings reveal several significant associations, including English proficiency, perceived financial need, frequency of communication with family, level of openness to family, and level of openness to friends. These results suggest that individuals with higher English proficiency, who have a positive perception of their financial situation, communicate frequently with family, and exhibit greater openness to both family and friends, have higher odds of reporting better mental health.

## 4. Discussion

In this exploratory analysis, we did not detect indications of the Vietnamese immigrant health paradox in the United States. While the finding contrasts with research suggesting that many foreign-born populations exhibit better health outcomes than their US-born counterparts despite socioeconomic disadvantage, it contributes to a growing body of literature that challenges the universality of the paradox and underscores the importance of subgroup-specific analyses. The existing literature [19,44,45,46,47], including meta-analyses [48,49], on the immigrant health paradox reveals a notable pattern. Despite facing socioeconomic disadvantages, many foreign-born populations tend to have better health outcomes than their US-born counterparts, especially in Western host countries and among recent arrivals. However, this advantage is not consistent across all groups. In addition, although several studies documented the Asian immigrant health paradox [19,23,50], emerging research has highlighted significant diversity among Asian-origin populations [51,52,53,54,55].

The absence of the immigrant health paradox among Vietnamese Americans in the United States can be explained by several factors. As a distinct immigrant group, they arrived in the US through various pathways, including refugee resettlement and family reunification. The first generation of Vietnamese primarily consists of individuals who were resettled as refugees following the Vietnam War, which involved forced displacement and prolonged trauma [56,57]. These experiences may diminish the “healthy migrant” effect often seen in voluntary economic migrants [58]. After migrating, Vietnamese immigrants face several challenges, including language barriers, discrimination in the labor market, and restricted socioeconomic mobility. These factors contribute to chronic stress and can undermine any health advantages typically associated with immigration [59]. Additionally, structural barriers such as poverty and limited access to culturally and linguistically appropriate healthcare further exacerbate these vulnerabilities [60,61]

Our exploratory findings indicate that certain factors related to migration and service experiences are associated with higher scores for depressive disorders, as assessed by the PHQ-2. Specifically, a longer duration of residence in the US, arriving at an older age, and frequent negative experiences with services all contribute to increased scores for depressive disorders. These results challenge acculturation models, suggesting that health declines with cultural assimilation [62,63]. A longer duration of residency in the U.S. may lead to cumulative exposure to systemic inequities and socioeconomic stress. Additionally, arriving at an older age often restricts labor market opportunities and complicates language integration. Frequent negative experiences with services can increase psychological strain, highlighting how structural and institutional barriers affect depression and mental health [64,65]. Consequently, a positive perception of financial situations not only reduces the severity of depressive disorders but is also associated with higher odds of reporting better physical and mental health. These findings underscore the importance of subjective economic well-being in promoting psychological resilience.

Our exploratory study found a pattern of factors associated with self-rated physical and mental health. In addition to a positive financial perception, higher English proficiency, greater openness, and more frequent communication with family and friends were associated with higher odds of reporting better physical and mental health. These exploratory findings demonstrate the protective effect of social connectedness within Vietnamese cultural norms and deepen the critical role of language in accessing services, employment, and social integration [66].

### 4.1. Limitations

This study offers insights into the relationship between nativity and various factors related to depressive disorder scores, as well as self-rated physical and mental health status among Vietnamese living in the US. However, our findings are subject to several limitations and should be interpreted with caution.

First, while the PHQ-2 is a widely used brief depression screener, the study did not assess measurement invariance between Vietnamese and U.S.-born respondents, limiting our ability to determine if it functions equivalently across cultures. Differences in how distress is expressed, norms around emotional disclosure, and stigma may affect the reporting of depressive symptoms, particularly among Vietnamese participants, potentially leading to response bias. Second, although validated Vietnamese translations of the PHQ exist, this study did not evaluate their linguistic or conceptual equivalence. Third, the PHQ-2’s brevity may exaggerate cultural differences in interpretation, as it captures only two symptoms that may not encompass all expressions of depression in certain cultural contexts.

Moreover, since this study uses a cross-sectional design, we cannot conclusively establish a causal relationship. Also, the survey is based on self-reported data, which cannot be independently verified and may introduce various biases. Our sample size is relatively small. Due to data inconsistencies, we excluded approximately 30% of responses from our analytical sample, potentially limiting statistical power and introducing uncertainty into our estimates. Furthermore, our modest sample size does not fully capture the diversity of the Vietnamese population in the US across geographic regions, migration waves, and generational cohorts, thereby limiting the generalizability of our findings. Another limitation of our analytical strategy is that estimating separate models to address multicollinearity may hinder our ability to evaluate the combined effects of correlated independent variables. While this approach is essential for obtaining stable coefficient estimates and avoiding inflated standard errors, it also restricts our capacity to draw inferences about how these variables interact within a single model.

Additionally, our study sample only includes individuals who identified as legal residents or US citizens. Notably, around 104,076 individuals—approximately 5% of the total Vietnamese population—live in the US without authorization [67]. Excluding this group from our analysis may introduce selective bias. Lastly, while we captured broad measures of financial stability, communication, and migration experiences, we did not collect detailed data on remittance obligations, debt burdens, the frequency and quality of social interactions, or the context of poor service experiences, all of which may significantly influence health outcomes.

Lastly, it is important to note that our study was conducted in early 2022, during a time when Asian communities, including Vietnamese populations, experienced a significant increase in public stigma and xenophobia due to the COVID-19 pandemic [68,69,70]. This environment likely influenced how respondents interpreted and reported experiences of discrimination. The heightened visibility of anti-Asian racism may have increased awareness of biased treatment for some individuals, while others may have become desensitized after prolonged exposure. Many participants may also have reinterpreted earlier interactions through the lens of the widely discussed anti-Asian hostility or felt a stronger sense of ethnic identity and solidarity, which affected how they expressed their experiences. Altogether, these factors suggest that our data reflect not only current events but also the lasting social and psychological effects of the pandemic’s earliest, most turbulent period.

### 4.2. Implications

Future studies should employ longitudinal and mixed-methods designs to examine causal pathways linking migration experiences, financial perception, social connectedness, language proficiency, and mental health. More detailed assurance of financial obligations, social network quality, and institutional experience will enhance the understanding of stressors and protective factors. Disaggregating data by migration wave, age at arrival, racialization, and legal status is essential. Policies promoting economic stability may improve the well-being of the Vietnamese population. Reducing barriers in healthcare and public service, including language access and culturally responsive communication, can mitigate depression associated with poor service experiences. Also, structural interventions should address cumulative disadvantages across socioeconomic, linguistic, and institutional domains. Clinical and community-based interventions should integrate approaches targeting financial stress, social support, and language proficiency. Programs strengthen family and peer communication, foster social integration, and provide trauma-informed care, which may also improve overall health. English language support, navigation assistance for services, and community health worker programs are recommended to reduce stressors associated with migration and systemic barriers. Interventions that address economic, social, and cultural determinants simultaneously are likely to be more effective in promoting health equity among Vietnamese living in the US.

## 5. Conclusions

Our findings that the immigrant health paradox is absent among Vietnamese immigrants highlight the need to move beyond one-size-fits-all narratives and adopt models that acknowledge the diversity within immigrant groups. Vietnamese immigrants illustrate how migration histories, structural barriers, and racialization influence health outcomes in ways that do not align with typical expectations. To address these disparities, it is essential to use disaggregated data, implement culturally relevant interventions, and develop policies that increase access to care and reduce structural inequities. By situating findings on Vietnamese immigrants within the broader context of the Asian American experience, this study underscores the need for precision in immigrant health research and the urgency of interventions that reflect the realities faced by diverse communities.

## Figures and Tables

**Table 1:** Participants’ Characteristics (N = 99).

Variable	Category or Median	n ^a^ or Mean	% or SD ^b^
Depressive disorders	No depressive disorder	25	26%
	Minor	32	32%
	Major	42	42%
Physical health status	Poor/Fair	8	8%
	Good	57	58%
	Very good	25	25%
	Excellent	9	9%
Mental health status	Poor/Fair	13	13%
	Good	47	47%
	Very good	24	24%
	Excellent	15	16%
Nativity	Non-US ^c^-born	64	65%
	US-born	35	35%
Gender	Male	33	33%
	Female	65	66%
	Other	0	0%
	Prefer not to say	1	1%
Age	<30 years	33	31%
	30–49 years	41	38%
	≥50 years	34	31%
Duration of living in the US	(years) Median = 28	25	±11
Age when arriving in the US	(years) Median = 19	22	±14
Marital Status	Never married	32	32%
	Married or living with a partner	60	61%
	Widowed	7	7%
	Divorced	0	0%
English proficiency	Not at all	1	1%
	Not well	13	13%
	Well	27	27%
	Very well	58	59%
Vietnamese proficiency	Not at all	5	5%
	Not well	20	20%
	Well	25	25%
	Very well	49	50%
Education level	Less than 12 years	2	2%
	12 years or high school graduate or GED ^d^	15	15%
	Post-high school training	3	3%
	Some college	18	18%
	College Graduate	41	42%
	Post-college graduate	19	20%
Employment status	Unemployed	5	5%
	Employed	75	76%
	Self-employed	8	8%
	Student	5	5%
	Retire	5	5%
	Disable	0	0%
	Other	1	1%
Income	(US dollars)	87 K ^e^	±66 K
Health Insurance	No health insurance	4	4%
	Private	69	70%
	Medicare	3	3%
	Government assisted	9	9%
	Tricare or military healthcare	6	6%
	VA ^f^	2	2%
	Other	6	6%
Perceived financial need	Not enough to meet the needs	9	9%
	Just enough to meet the needs	64	67%
	More than enough to meet the needs	23	24%
Frequency of being treated with less respect.	Less than once a year	42	44%
	A few times a year	34	36%
	A few times a month	14	15%
	At least once a week	4	4%
	Most everyday	1	1%
Frequency of receiving poorer service.	Less than once a year	46	48%
	A few times a year	35	37%
	A few times a month	10	11%
	At least once a week	3	3%
	Most everyday	1	1%
Frequency of being threatened or harassed.	Less than once a year	72	76%
	A few times a year	14	15%
	A few times a month	5	5%
	At least once a week	2	2%
	Most everyday	2	2%
Frequency of communication with family or relatives (other than spouse/partner)	Less than once a year	14	14%
	A few times a year	10	10%
	A few times a month	37	39%
	At least once a week	23	24%
	Most everyday	13	13%
Openness level to family or relatives (other than spouse/partner)	Not at all	19	20%
	A little	32	33%
	Some	31	32%
	Lot	15	15%
Reliability on family or relatives (other than spouse/partner)	Not at all	21	22%
	A little	18	18%
	Some	32	33%
	Lot	26	27%
Frequency of communication with friends	Less than once a year	27	28%
	A few times a year	6	6%
	A few times a month	30	31%
	At least once a week	18	19%
	Most everyday	16	16%
Openness level to friends	Not at all	19	19%
	A little	27	28%
	Some	32	33%
	Lot	19	20%
Reliability on friends	Not at all	24	25%
	A little	23	25%
	Some	27	28%
	Lot	23	24%
Difficulty finding a job due to Vietnamese descent.	No	78	80%
	Yes	19	20%

^a^ n, count (total number that does not add to N = 99 indicates missing responses); ^b^ SD, standard deviation; ^c^ US, the United States; ^d^ GED, General Educational Development; ^e^ K, in thousands; ^f^ VA, Veteran Affairs.

**Table 2:** Linear regression output for the relationship between depressive disorders and exploratory factors. (N = 99).

Model	Independent Variables	Coefficient	95% CI ^a^	*p*-Value
Nativity	Nativity	0.501	−0.139–1.142	0.123
Demographic characteristics & Socioeconomic status	Gender	0.634	−0.230–1.450	0.146
	Age	−0.456	−0.107–0.016	0.142
	Marital Status	−0.853	−1.965–0.258	0.129
	**Duration of living in the US**	**0.061**	**0.007–0.115**	**0.027**
	**Age when arriving in the US**	**0.100**	**0.026–0.174**	**0.009**
	Vietnamese proficiency	−0.363	−1.096–0.368	0.323
	English proficiency	−0.430	−1.041–0.181	0.164
	Education level	0.174	−0.115–0.464	0.231
	Employment	−0.032	−0.849–0.785	0.937
	Income	−0.001	−0.007–0.004	0.640
Material factors	Health insurance	−0.098	−0.640–0.443	0.720
	**Perceived financial need**	**−1.440**	**−1.896–−0.985**	**<0.001**
Psychological factors	Frequency of communication with family or relatives other than spouse/partner	−0.135	−0.393–0.124	0.302
	Openness level to family or relatives other than spouse/partner	−0.341	−0.691–0.008	0.055
	Reliability on family or relatives other than spouse/partner	0.101	−0.227–0.430	0.541
	Frequency of communication with friends	0.032	−0.200–0.265	0.783
	Openness level to friends	−0.279	−0.638–0.081	0.127
	Reliability on friends	−0.175	−0.534–0.184	0.335
	Frequency of being treated with less respect	−0.016	−0.484–0.451	0.946
	**Frequency of receiving poorer service**	**0.706**	**0.185–1.228**	**0.009**
	Frequency of being threatened or harassed	0.091	−0.304–0.487	0.648

^a^ CI, confidence interval; Boldface indicates significance at 95% (*p*-value < 0.05).

**Table 3:** Ordinal logistic regression output for the odds of factors associated with self-rated physical health. (N = 99).

Model	Independent Variables	OR ^a^	95% CI ^b^	*p*-Value
Nativity	Nativity	1.443	0.647–3.220	0.371
Demographic characteristics & Socioeconomic status	Gender	2.651	0.546–12.872	0.226
	Age	1.226	0.878–1.711	0.230
	Marital Status	0.168	0.016–1.747	0.136
	Duration of living in the US ^c^	0.819	0.604–1.110	0.199
	Age when arriving in the US ^c^	0.830	0.587–1.174	0.293
	Vietnamese proficiency	3.470	0.668–18.015	0.139
	**English proficiency**	**8.608**	**2.241–33.063**	**0.002**
	Education level	1.126	0.646–1.962	0.675
	Employment	3.610	0.575–22.642	0.171
	Income	1.003	0.991–1.014	0.630
Material factors	Health insurance	1.328	0.538–3.278	0.538
	**Perceived financial need**	**4.566**	**2.122–9.822**	**<0.001**
Psychological factors	**Frequency of communication with family or relatives other than spouse/partner**	**1.558**	**1.040–2.334**	**0.031**
	Openness level to family or relatives other than spouse/partner	1.089	0.647–1.830	0.748
	Reliability on family or relatives other than spouse/partner	1.053	0.643–1.721	0.838
	Frequency of communication with friends	0.723	0.506–1.035	0.077
	**Openness level to friends**	**1.878**	**1.078–3.270**	**0.026**
	Reliability on friends	1.140	0.684–1.900	0.614
	Frequency of being treated with less respect	0.889	0.439–1.800	0.744
	Frequency of receiving poorer service	0.578	0.264–1.264	0.170
	Frequency of being threatened or harassed	1.162	0.649–2.079	0.613

^a^ OR, odds ratio; ^b^ CI, confidence interval; ^c^ US, United States; Boldface indicates significance at 95% (*p*-value < 0.05).

**Table 4:** Ordinal logistic regression output for the odds of factors associated with self-rated mental health. (N = 99).

Model	Independent Variables	OR ^a^	95% CI ^b^	*p*-Value
Nativity	Nativity	0.544	0.242–1.220	0.140
Demographic characteristics & Socioeconomic status	Gender	0.407	0.116–1.421	0.159
	Age	1.174	0.900–1.534	0.237
	Marital Status	3.039	0.552–16.739	0.202
	Duration of living in the US ^c^	0.848	0.664–1.084	0.189
	Age when arriving in the US ^c^	0.868	0.659–1.146	0.321
	Vietnamese proficiency	2.325	0.639–8.458	0.200
	**English proficiency**	**3.345**	**1.247–8.975**	**0.016**
	Education level	1.114	0.699–1.777	0.649
	Employment	1.058	0.280–3.989	0.934
	Income	1.004	0.995–1.013	0.307
Material factors	Health insurance	1.282	0.560–2.933	0.557
	**Perceived financial need**	**7.611**	**3.411–16.982**	**<0.001**
Psychological factors	**Frequency of communication with family or relatives other than spouse/partner**	**1.855**	**1.204–2.859**	**0.005**
	**Openness level to family or relatives other than spouse/partner**	**3.180**	**1.764–5.732**	**<0.001**
	Reliability on family or relatives other than spouse/partner	0.875	0.526–1.455	0.607
	Frequency of communication with friends	0.804	0.561–1.153	0.236
	**Openness level to friends**	**2.415**	**1.352–4.313**	**0.003**
	Reliability on friends	0.750	0.437–1.287	0.296
	Frequency of being treated with less respect	0.670	0.318–1.409	0.291
	Frequency of receiving poorer service	0.444	0.186–1.063	0.069
	Frequency of being threatened or harassed	0.964	0.525–1.772	0.906

^a^ OR, odds ratio; ^b^ CI, confidence interval; ^c^ US, United States; Boldface indicates significance at 95% (*p*-value < 0.05).

## Data Availability

The data presented in this study are available on request from the corresponding author (The data are not publicly available due to privacy restrictions).

## Supplementary Materials

The following supporting information can be downloaded at: https://www.mdpi.com/article/10.3390/healthcare14030354/s1, Survey Instrument.
