# Supplementary material for: Exploring the Immigrant Health Paradox Among the Vietnamese Population in the United States
Source: Healthcare (Basel). 2026 Jan 30;14(3):354. doi: 10.3390/healthcare14030354 (PMC12896797; doi:10.3390/healthcare14030354)
Supplement: Supplementary file 1 [file healthcare-14-00354-s001.zip › healthcare-4080916-supplementary.pdf]

Is Vietnamese your origin or ancestry?	0	No
	1	Yes
Are you a citizen or permanent resident of the US?	0	No
	1	Yes
Are you at least 18 years of age as of today?	0	No
	1	Yes
**If there is any answer of NO [0]**	**Thank you for your interest in this study. (STOP)**	
What is your gender?	0	Male
	1	Female
	2	Other
	3	Prefer not to Answer
what year were you born?	Four digits number	
Were you born in the United States (US)?	0	No
	1	Yes
If you were not born in the United States, how long have you lived in the US? (in years)	Continuous numbers	
How old were you when you moved to the US? (in years)	Continuous numbers	
What state do you live?	Free text	
What is your marital status?	0	Never married
	1	Married or Living with a partner
	2	Widowed
	3	Divorced
Was either or were both of your parents born in the US?	0	No
	1	Yes
How well do you speak Vietnamese?	0	Not at all
	1	Not well
	2	Well
	3	Very well
How well do you speak English?	0	Not at all (to the next 2 questions)
	1	Not well (to the next 2 questions)
	2	Well (skip the next 2 questions)
	3	Very well (skip the next 2 questions)
Do you find it hard interacting with others because of difficulties you have with the	0	No
	1	Yes
Do people treat you badly because they think you do not speak English well or	0	No
	1	Yes
What is the highest grade or level of schooling you completed?	0	Less than 12 years
	1	12 years or High School or GED
	2	Post high school training (vocational or technical)
	3	Some college
	4	College Graduate

	5	Post College Graduate
What is your current occupational status?	0	Unemployed
	1	Employed
	2	Self-employed
	3	Student
	4	Retire
	5	Disabled
	6	Other
Is it difficult to find work due to Vietnamese descent?	0	No
	1	Yes
What kind of health insurance do you have?	0	no health insurance
	1	Private (through an employer or union; purchased directly from an insurance company)
	2	Medicare, for people 65 and older, or people with certain disabilities
	3	Medicaid, Medical Assistance, or any kind of government-assistance plan for those with low incomes or a disability
	4	TRICARE or other military health care
	5	VA (including those who have ever used or enrolled for VA health care)?
	6	Other
What is your best estimate of your annual household income before taxes and deductions from ALL jobs and businesses?	Continuous numbers	
In general, would you say that you or your family have/has the money:	0	Not enough to meet your needs
	1	Just enough to meet your needs
	2	more than enough to meet your needs
How would you rate your overall physical h	0	Poor/Fair
	1	Good
	2	Very good
	3	Excellent
How would you rate your overall mental he	0	Poor/Fair
	1	Good
	2	Very good
	3	Excellent
The below questions ask about your last 2 weeks. How often have you been bothered by any of the following problems?		
Little interest or pleasure in doing things?	0	Not at all
	1	Several days
	2	More than half the days
	3	Nearly every day
	0	Not at all

Feeling down, depressed, or hopeless?	1	Several days
	2	More than half the days
	3	Nearly every day
**The below questions ask about your social networking among friends and family.**		
How often do you talk on the phone or get together with family or relatives who do not live with you? (Not include your spouse/partner)	0	Less than once a month
	1	Once a month
	2	A few times a month
	3	A few times a week
	4	Most every day
How much can you open up to relatives who do not live with you if you need to talk about your worries? (Not include your spouse/partner)	0	Not at all
	1	a little
	2	some
	3	Lot
How much can you rely on relatives who do not live with you for help if you have a serious problem? (Not include your spouse/partner)	0	Not at all
	1	a little
	2	some
	3	Lot
How often do you talk on the phone or get together with friends?	0	Less than once a month
	1	Once a month
	2	A few times a month
	3	A few times a week
	4	Most every day
How much can you open up to friends and talk about worries?	0	Not at all
	1	a little
	2	some
	3	Lot
How much can you rely on your friends for help if you have a serious problem?	0	Not at all
	1	a little
	2	some
	3	Lot
**The below questions ask about your daily experience.**		
Frequency treated w/ less respect than others?	0	Less than once a year
	1	A few times a year
	2	A few times a month
	3	At least once a week
	4	Most every day
Frequency receive poorer service than others?	0	Less than once a year
	1	A few times a year
	2	A few times a month
	3	At least once a week
	4	Most every day
	0	Less than once a year
	1	A few times a year

Frequency threatened/harassed?	2	A few times a month
	3	At least once a week
	4	Most every day
**Thank you for your pariticipating in this study**		
